# Supplementary material for: Nucleotide depletion reveals the impaired ribosome biogenesis checkpoint as a barrier against DNA damage
Source: EMBO J. 2020 Jun 2;39(13):e103838. doi: 10.15252/embj.2019103838 (PMC7327477; doi:10.15252/embj.2019103838)
Supplement: Supplementary file 1 — Appendix [file EMBJ-39-e103838-s001.pdf]

Appendix for

Nucleotide depletion reveals the impaired ribosome biogenesis checkpoint as a barrier against DNA damage

by

Pelletier, Riaño-Canalias et al.

Table of Contents:

Appendix Figure Legends S1-S6

Appendix Figures S1-S6

Appendix Tables S1-S3

Appendix Figure S1. Severe and acute inhibition of guanosine nucleotide synthesis induces replicative stress but not DNA double strand breaks

A, Western blots showing the levels of the indicated protein in HCT116 cells treated for 8, 16, or 24 hrs with the indicated concentrations of MPA. GAPDH served as a loading control.

B, HCT116 cells were treated for 24 hrs with the vehicle alone (-),10 µM MPA (MPA) or 50 µM etoposide (Eto), before cell fixation and immunostaining of γ-H2AX (green) and 53BP1 (red). DNA was counterstained with DAPI (blue). One z-confocal plane is shown. Scale bars correspond to 10 µm.

C-D Proportion of cells with the indicated number of foci (0; 1-4; >4) after immunostaining of γ-H2AX (**C**) or 53BP1 (**D**) from (B). Quantification was performed on 150-300 cells for each condition. ****p<0.0001 by two tail T-Student's test. A.U. Arbitrary Units.

E, Western blots showing the level of the specific proteins in HCT116 cells treated for 24 hrs with the indicated concentration of AVN944. GAPDH was used as a loading control.

F, Western blots showing the level of the specific proteins in HCT116 cells treated for 24 hrs with vehicle (-), 10 µM MPA (MPA), 10 µM AVN944 (AVN944) or 50 µM etoposide (Eto).

G-J, HCT116 cells were treated for 24 hrs with the vehicle alone (-), 1 µM MPA, 10 µM MPA, or the combination of 10 µM MPA with 400 µM guanosine, and the levels of IMP (**G**) adenylate nucleotides (**H**), UTP (**I**), and NAD^+ and NADH (**J**), plotted as the ratio NAD^+/NADH, were analyzed as in (Fig 1B). Mean ± SEM are representative of 3 independent experiments carried out in triplicate.

K-N, HCT116 stable cells expressing an independent tetracycline-inducible shRNA against IMPDH2 and a NT shRNA ($IM2^{iKD\#2}$-shNT) were treated as in (Fig 1C). **K,** IMPDH1, and IMPDH2 mRNA levels were quantified by real-time qPCR in 2 independent experiments in triplicate and normalized to β-Actin mRNA. **L,** Whole cell extracts were analyzed on Western blots with the indicated antibodies. GAPDH was used as a loading control. **M,** Same clone was treated in the absence or presence of doxycycline (dox) (2 µg/mL) for 6 days, followed by a 24 hrs treatment with 10 µM MPA. Whole cell extracts were analyzed on Western blots with the indicated antibodies, GAPDH was used as a loading control, and band intensity of p53 and p21 were quantified. Mean ± SD of at least two independent experiments are shown (right

panel). **N,** The levels of the indicated guanosine nucleotides were measured in technical triplicates by LC-MS, normalized to protein content and presented as Mean ± SEM.

O, Western blots showing the levels of p53 in HCT116 cells treated for 24 hrs with 10 µM MPA, in the presence of DMSO (-) or the indicated nucleoside (400 µM). α-tubulin served as a loading control. G, Guanine; A, Adenine; U, Uracil; C, Cytosine.

Data information: In panels G-K, and M-N, data are presented as relative to control. *p<0.05, **p<0.01, ***p<0.001, by two tail T-Student's test.

Appendix Figure S2. Validation of RPL11 depletion and ATR/ATM inhibition

A, HCT116 cells were transfected and treated as in (Fig 2A). RPL11 mRNA levels were quantified by real-time qPCR in triplicate, and normalized to β-actin mRNA.

B, HCT116 cells were transfected with either a NT or a siRNA against RPL11 (siRPL11); RPL7a (siRPL7a) or the combination of both (siRPL11 + siRPL7a) for 24 hrs as in (Fig 2B) and cells were collected before addition of the treatments. The levels of RPL11 and RPL7a were analyzed by real-time qPCR and normalized to β-actin mRNA. Results are representative of two independent experiments carried out in triplicate.

C, HCT116 cells were pretreated 30 mins with the vehicle alone (-), 10 µM of the ATR inhibitor VE-821 (ATRi), 10 µM of the ATM inhibitor KU-55933 (ATMi), or the combination of both drugs and treated with vehicle (-) or 10 µM MPA in the continued absence or presence of ATRi/ATMi for additional 24 hrs. The indicated proteins were analyzed on Western blots. GAPDH was used as a loading control.

D, HCT116 cells were transfected with either a NT or a siRNA against RPL11 (siRPL11) as in (Fig 2C) and cells were collected before addition of the treatments. RPL11 mRNA was analyzed by real-time qPCR and normalized to β-actin mRNA. Results are representative of two independent experiments carried out in triplicate.

Data information: In panels A, B, and D, Mean ± SD are presented as relative to siNT. ***p<0.001, by two tail T-Student's test.

Appendix Figure S3. Ribosome biogenesis and the formation of the IRBC complex are not further altered by higher concentrations of MPA

A, HCT116 cells were treated with either vehicle alone (-), 1 µM MPA, 10 µM MPA or 5 nM ActD for 24 hrs. After lysis, an exogenous firefly luciferase mRNA spike was

added proportionally to the DNA content. The levels of Internal Transcribed Spacer (ITS) 1 and ITS2 contained in the 47S rRNA, SDH-B, c-Myc and IMPDH2 mRNAs were measured by real-time qPCR, normalized by luciferase mRNA. Results are representative of 2 independent experiments carried out in triplicate.

B, HCT116 cells were transfected with Renilla Luciferase and 24 hrs later treated with either vehicle alone (-), 1 µM MPA, 10 µM MPA, or 10 µg/mL α-amanitin (α-am) for 24 hrs. The firefly luciferase activity was measured, normalized to protein concentration. Results are representative of 3 independent experiments carried out in triplicate.

C, HCT116 cells were treated as in (A) followed by cell fixation and immunostaining of UBF (green) and Fibrillarin (red). DNA was counterstained with DAPI (blue). One z-confocal plane is shown. Scale bars correspond to 5 μm.

D, HCT116 cells were treated with 10 µM MPA for the indicated time or with 100 µg/mL cycloheximide (CHX) for 1 hr. The rate of global protein synthesis was quantified by incorporation of ^{3}H-leucine into nascent protein. Results were normalized to the concentration of total protein. Results are representative of 3 independent experiments carried out in triplicate.

E-F, HCT116 cells were treated as in (Fig 3D). Cells lysates were collected, subjected to ultracentrifugation, spiked with firefly luciferase mRNA and immunoprecipitated with anti-RPL5 antibody (IP-RPL5) or the IgG control (IgG). **E,** Levels of the indicated proteins were analyzed on Western blots in the post-ribosomal lysates (INPUT, left panel) or in the immunoprecipitated fraction (right panel). The results are representative of 2 independent experiments. **F,** The levels of 5S rRNA co-immunoprecipitated with RPL5 were determined by real-time qPCR and normalized to the firefly luciferase mRNA. Data, representative of 3 independent experiments carried out in triplicate, are presented as relative to untreated cells immunoprecipitated with anti-RPL5.

G, p53 proteins levels from (Fig 3G) were quantified and normalized to GAPDH in at least four independent experiments. P values are indicated on the graph.

Data information: In panels A, B, D, F, and G, Mean ± SD are presented as relative to control. *p<0.05, **p<0.01, ***p<0.001, by two tail T-Student's test.

Appendix Figure S4. Higher concentrations of MPA lead to S-phase entry, inhibition of DNA replication and progressive induction of DNA damage.

A, Percentage of cells in each cell cycle phase from (Fig 4A) were quantified in at least 3 independent experiments, and presented as Mean ± SD. **p<0.01, ***p<0.001, by two tail T-Student's test.

B, HCT116 cells were subjected to serum deprivation for 16 hrs, followed by the re-addition of serum in the absence or presence of 10 µM MPA for either 6 or 24 hrs, propidium iodide staining and FACS analysis.

C, HCT116 cells were serum-deprived for 16 hrs, treated with 10 µM MPA with serum for the indicated times and then subjected to propidium iodide staining and FACS analysis.

D, Cell cycle profiles corresponding to the data shown in (Fig 4B).

E, Western blots from LoVo, LS174, RKO, and HCT116 cell lines treated for 24 hrs with vehicle alone (-),1 µM MPA or 10 µM MPA.

F, HCT116 cells treated as in (Fig 4A) were analyzed for the indicated proteins. GAPDH was used as a loading control.

G, Scheme of the labeling protocol. HCT116 cells were pretreated with the vehicle alone (-) or 10 µM MPA for 2 or 23 hrs, and then in the continued presence of MPA, the indicated nucleic acid analogues were added and DNA fibers were prepared and labeled with anti-BrdU antibodies (see Methods).

H, Proportion of cells with the indicated number of foci (0; 1-5; >5) positive for both RPA and BrdU under native staining conditions quantified from (Fig 4E). Quantification was performed on 150-200 cells for each biological replicate (n=3), and presented as mean ± SD. **p<0.01, ***p<0.001,****p<0.0001 by two tail T-Student's test.

I, HCT116 cells were treated with either the vehicle alone (-) or 50µM etoposide (Eto) before γ-H2AX immuno-labeling, propidium iodide staining and FACS analysis. The total intensity of γ-H2AX and propidium iodide were determined in 20,000 cells and plotted in a scatter diagram. Negative represents etoposide treated cells without γ-H2AX primary antibody.

J, HCT116 cells were treated as in (C) and subjected to γ-H2AX immuno-labeling, propidium iodide staining and FACS analysis. Cell cycle profiles of the total cell population (upper panel, left), the total intensity of γ-H2AX and propidium iodide (second line panel) and the specific cell cycle profiles of γ-H2AX^{high} (third line panel) and γ-H2AX^{low} (lower panel) cell populations are shown. The dotted lines represent the G1 (1n) and G2-M (2n) average cell populations

Appendix Figure S5. Absence of p21 favors S-phase accumulation at high concentrations of MPA.

A, Isogenic HCT116 wild-type (wt) or HCT116 p21$^{-/-}$ cells were treated for 24 hrs with vehicle alone (-) or 10 µM MPA, and subjected to propidium iodide staining and FACS analysis. Percentage of cells in each cell cycle phase was analyzed in 3 independent experiments and presented as mean ± SD (right panel). *p<0.05, **p<0.01, ***p<0.001, by two tail T-Student's test.

B, HCT116 cells from (Fig. 5B), were lysed and p53 and p21 protein levels analyzed on Western blots. GAPDH was used as a loading control.

Appendix Figure S6. The downregulation of RPL5 and 5S rRNA, but not of RPS6, enhances MPA-mediated DNA-damage.

A, Percentage of cells in each cell cycle phase from (Fig 6A). mean ± SD are representative of at least 3 independent experiments.

B, Cells were treated as in (Fig 6A) and subjected to BrdU labeling (see Material and Methods), propidium iodide staining and FACS analysis. The results are representative of two independent experiments.

C, HCT116 cells were transfected with siRNAs against NT, RPL11, RPL5 or RPS6 and were collected before treatment, 24 hrs later. mRNA levels of RPL11 (left panel), RPL5 (middle panel) or RPS6 (right panel) were analyzed by real-time qPCR, normalized to β-actin mRNA, and presented as relative to siNT. Mean ± SD are representative of three independent experiments carried out in triplicate.

D, HCT116 cells were transfected with siRNAs against NT, RPL5 or RPS6 and 24 hrs later were treated or not with 10 µM MPA for 72 hrs. Indicated proteins were analyzed on Western blots and GAPDH was used as a loading control.

E, Statistical analysis of the immunostaining of γ-H2AX in HCT116 cells transfected with a siRNA against NT or RPL5 for 24 hrs and treated with 10 µM MPA for 72 hrs when indicated. n>1900 cells, from 2 independent experiments.

F, Representative images from (E) of cells labeled with γ-H2AX (green) and DAPI (blue).

G Statistical analysis of the immunostaining of γ-H2AX in HCT116 cells treated as in (E) with an siRNA against NT or RPS6. n>1400 cells, from 3 independent experiments.

H, Representative images from (G) of cells labeled with γ-H2AX (green) and DAPI (blue).

I, HCT116 cells were transfected three consecutive days with a siRNA against NT or TIFIIIa. mRNA levels of TIFIIIa were analyzed by real-time qPCR in technical triplicate, normalized to β-actin mRNA and presented as mean ± SD, relative to siNT. *$p<0.05$, by two tail T-Student's test.

J, HCT116 cells transfected as in (I) were pulse labeled with ^{3}H-Uridine for 2 hrs, and chased in non-radioactive media for 4 hrs. 18S and 28S rRNAs were analyzed on autoradiogram (upper panel) and Ethidium Bromide (EtBr)-stained agarose gel (lower panel).

K, HCT116 cells were transfected as in (i) and treated or not with 10 μM MPA for 72 hrs. The indicated proteins were analyzed on Western blots and GAPDH was used as a loading control.

L, Statistical analysis of the immunostaining of γ-H2AX. $n>1000$ cells, from 1 experiment.

M, Representative images from (K) of cells labeled with γ-H2AX (green) and DAPI (blue).

Data information: In panels E, G and L, data are presented as mean ± SD. ***$p<0.001$, ****$p<0.0001$, by Kruskal-Wallis test. In panels C and I, *$p<0.05$, ***$p<0.001$ by two tail T-Student's test. A.U. Arbitrary Units. Scale bars in F, H and M correspond to 10 μm.

Supplementary Figure 1.

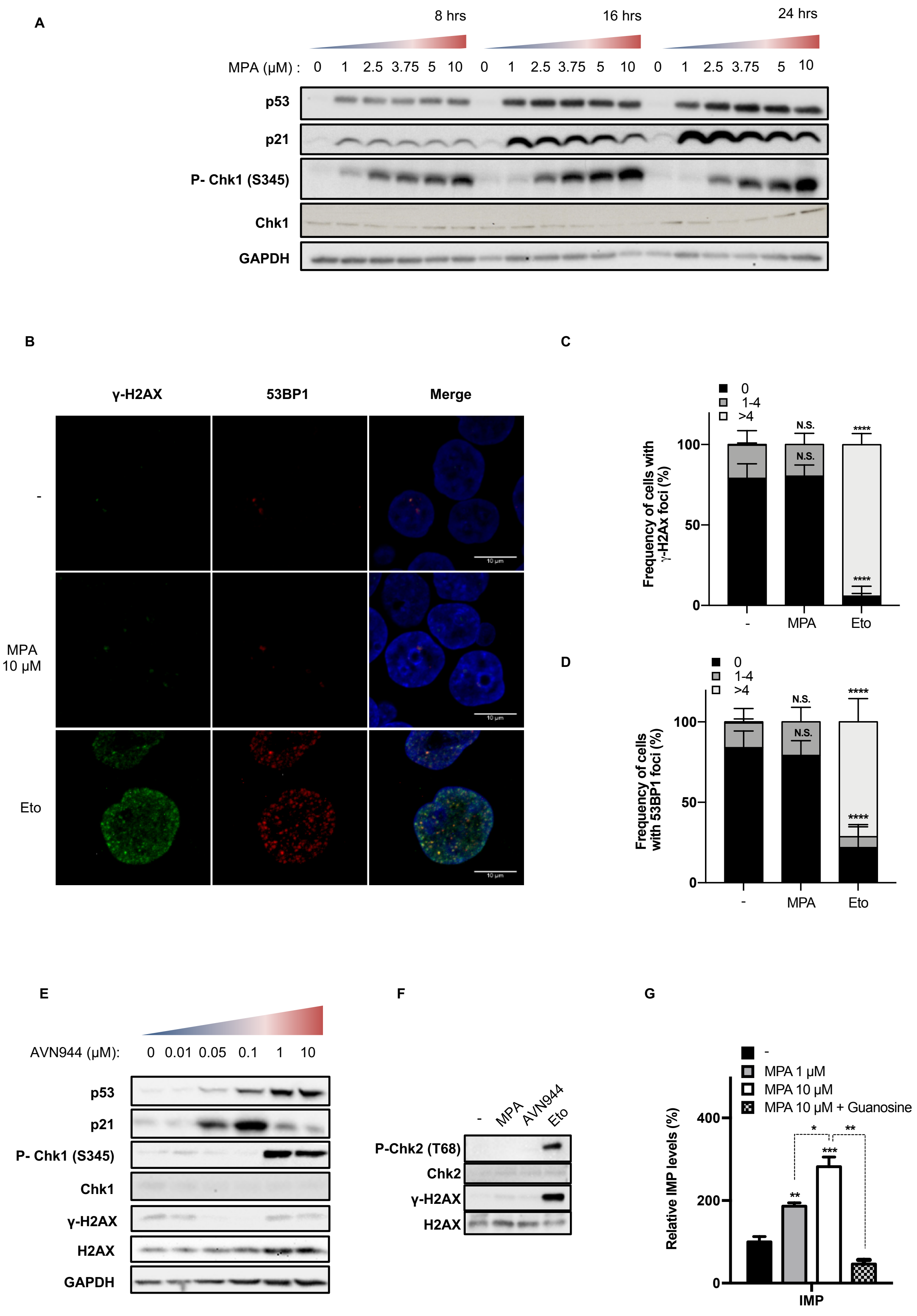

Supplementary Figure 1.

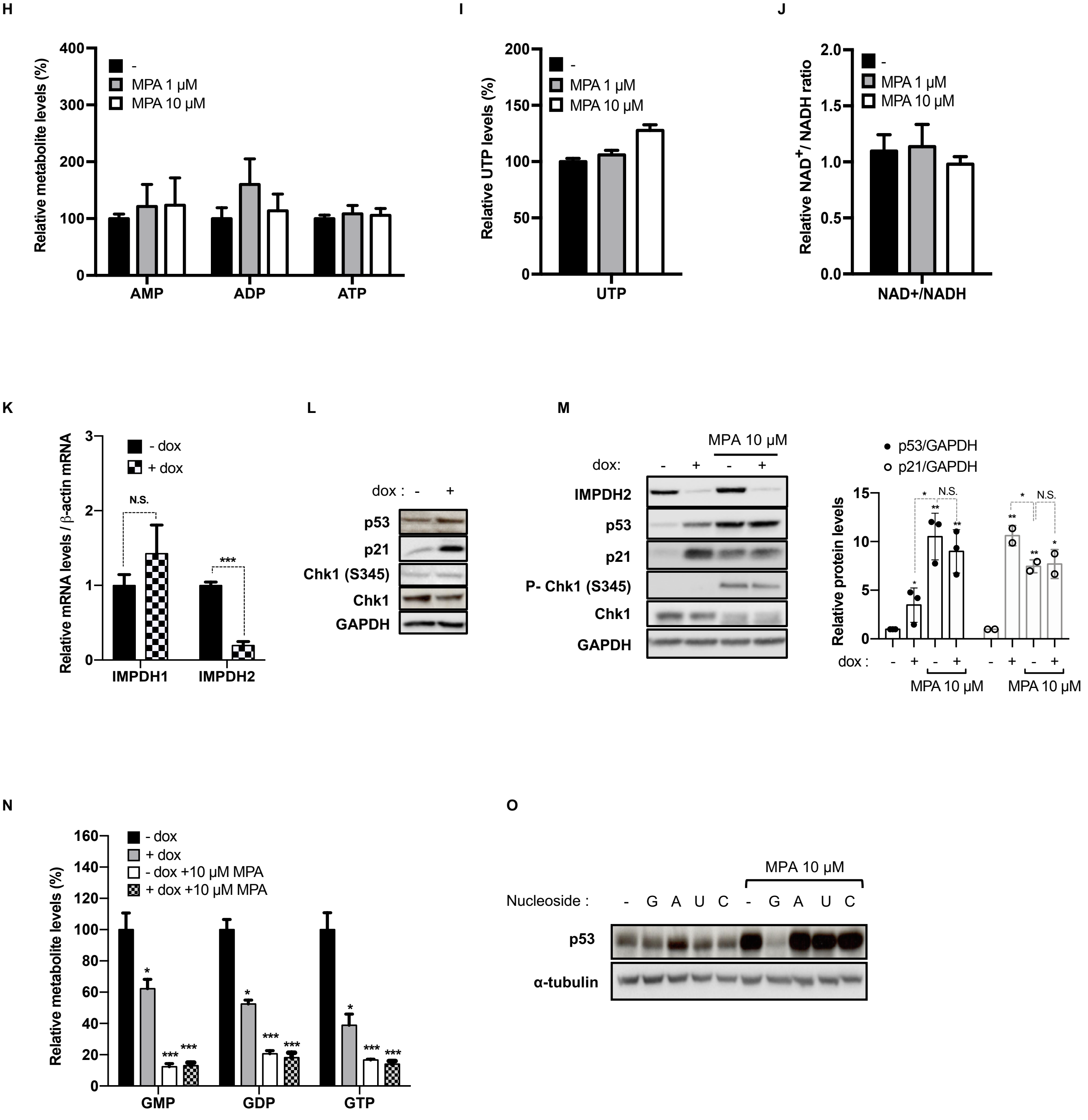

Supplementary Figure 2.

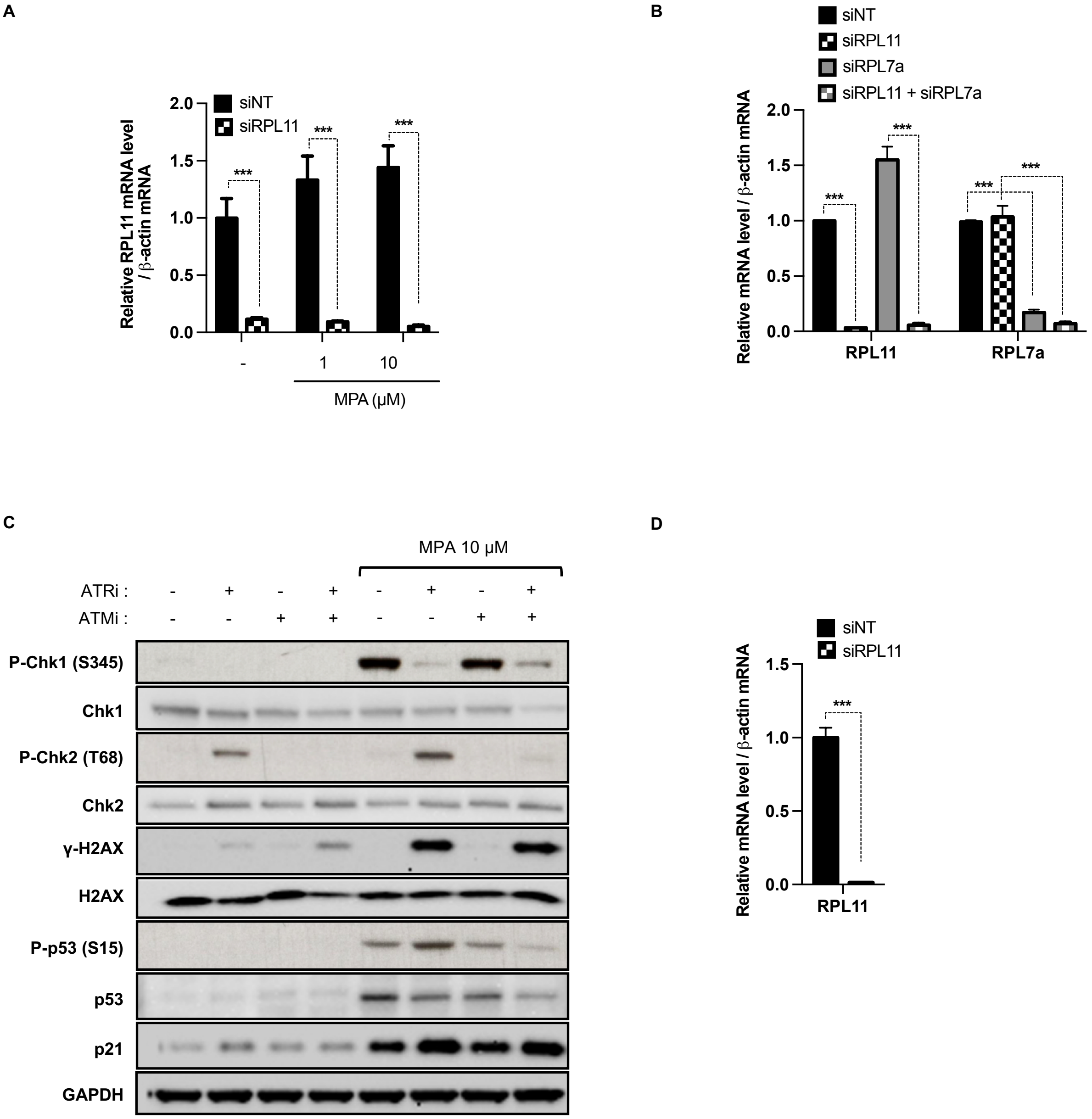

Supplementary Figure 3.

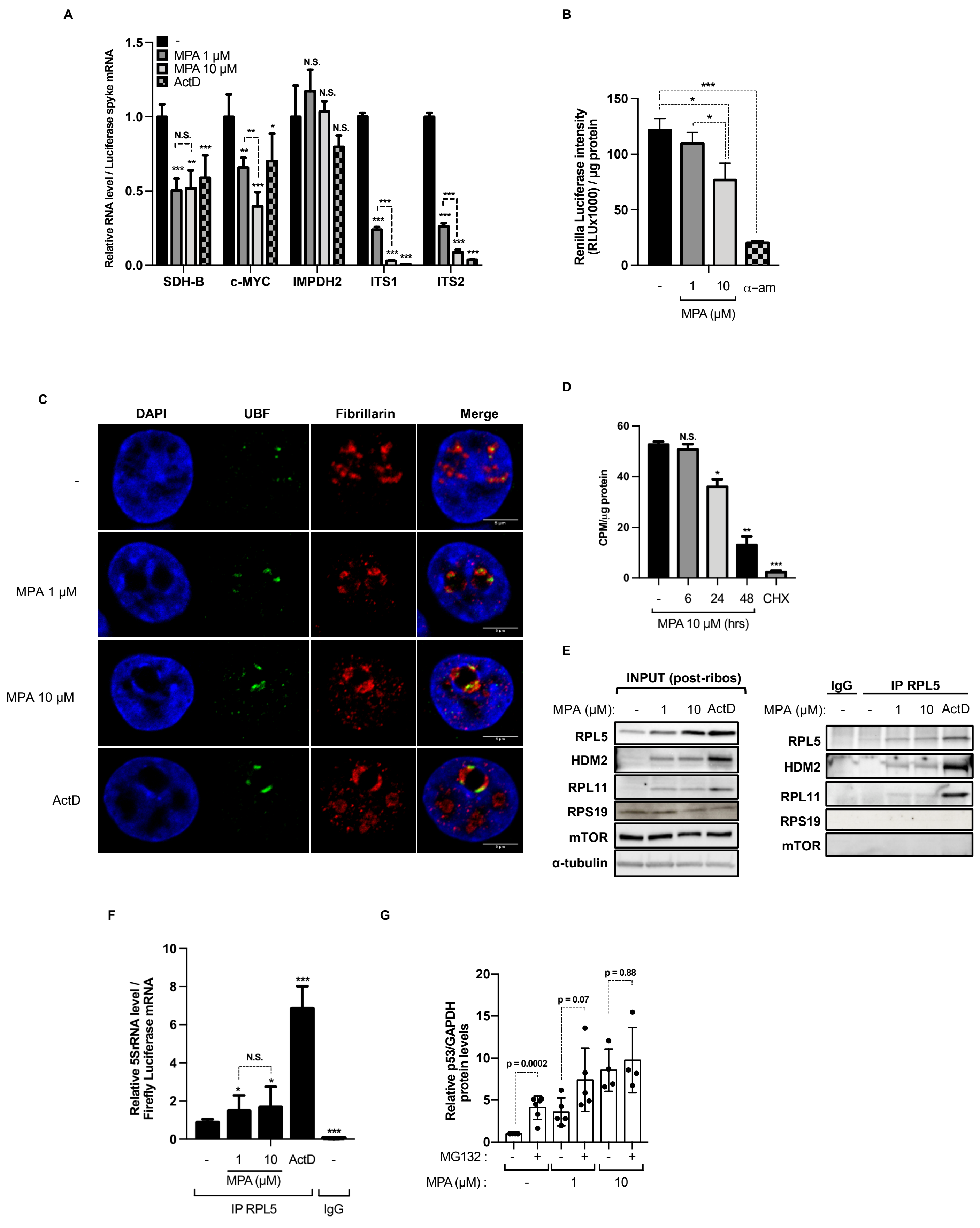

Supplementary Figure 4.

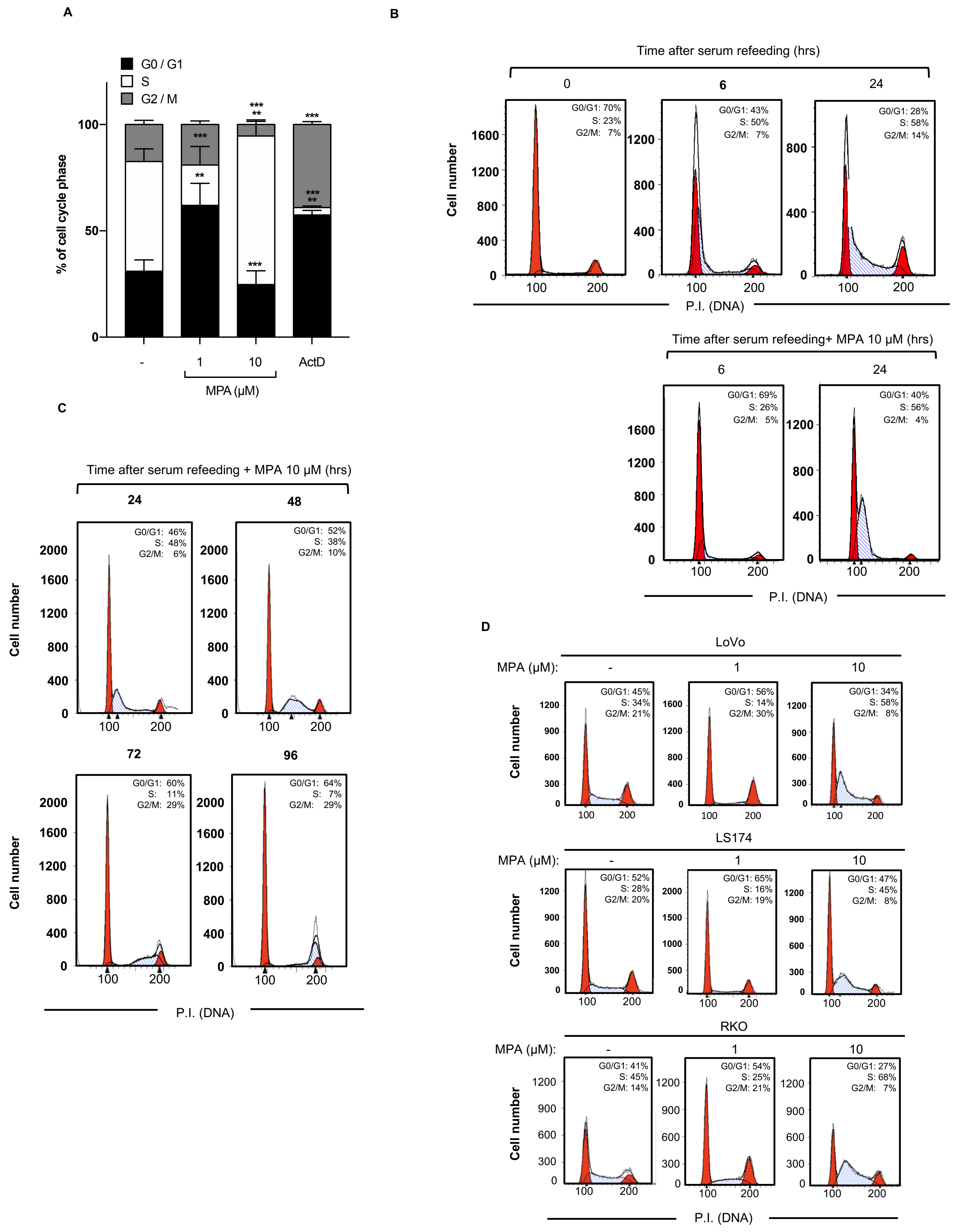

Supplementary Figure 4

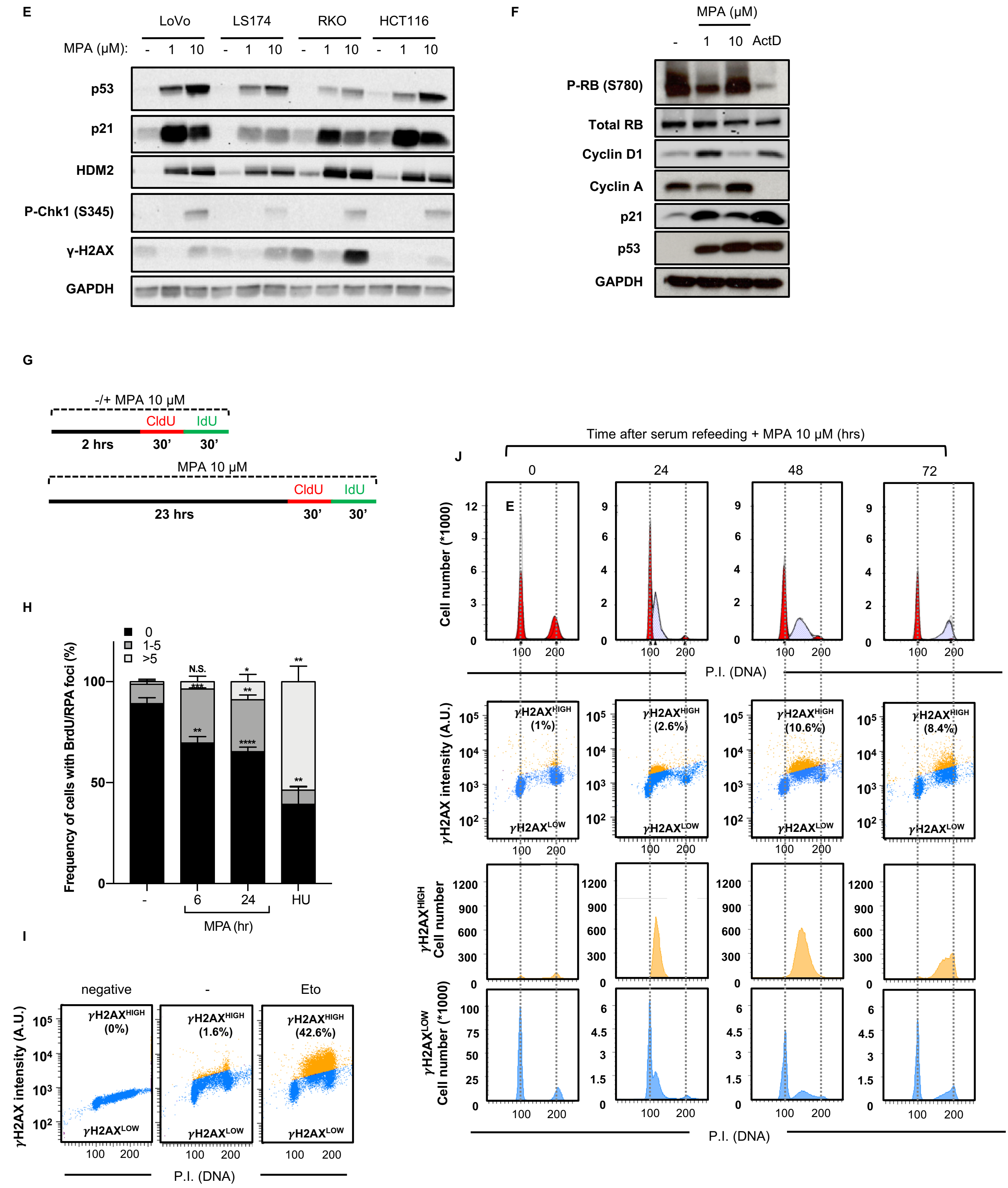

Supplementary Figure 5

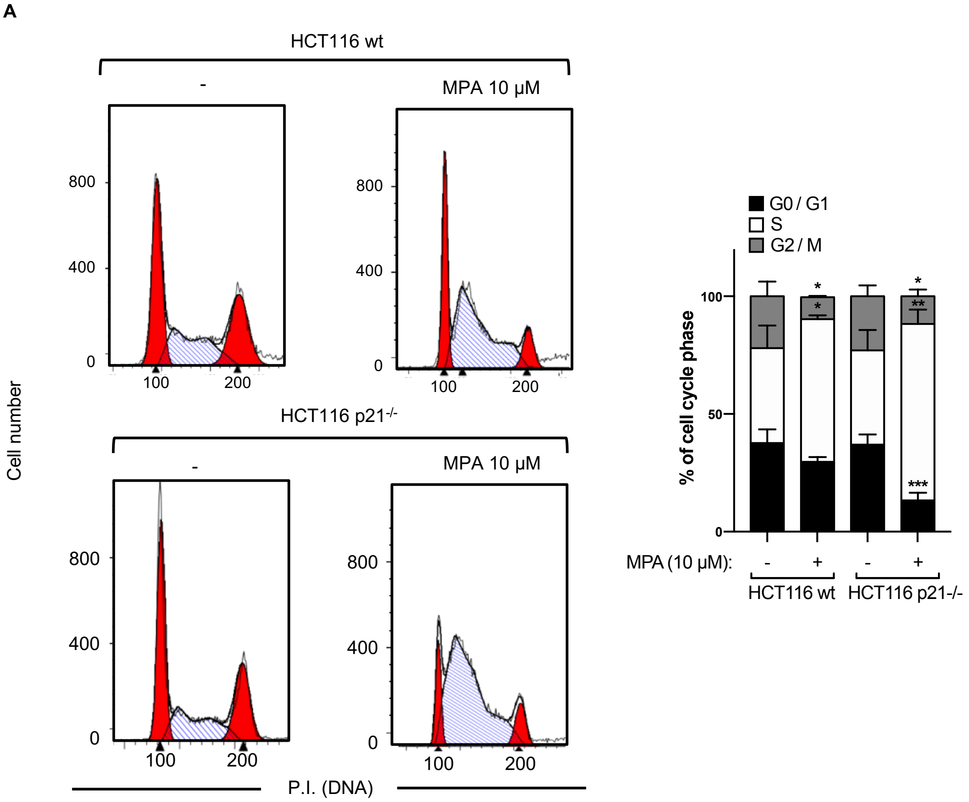

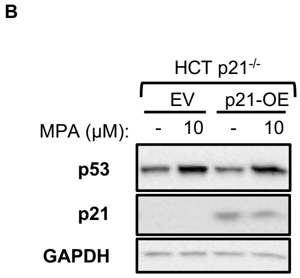

Supplementary Figure 6

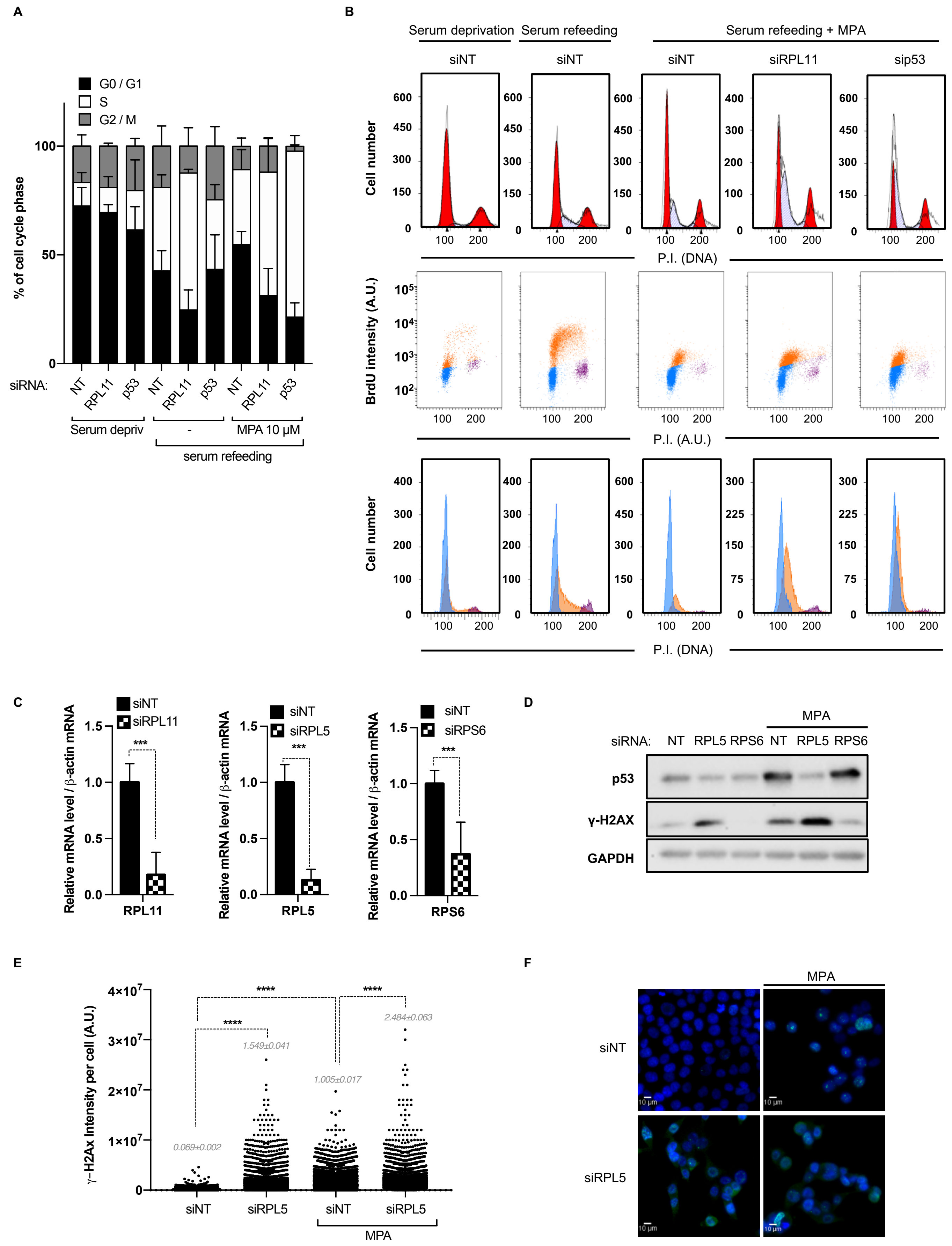

Supplementary Figure 6

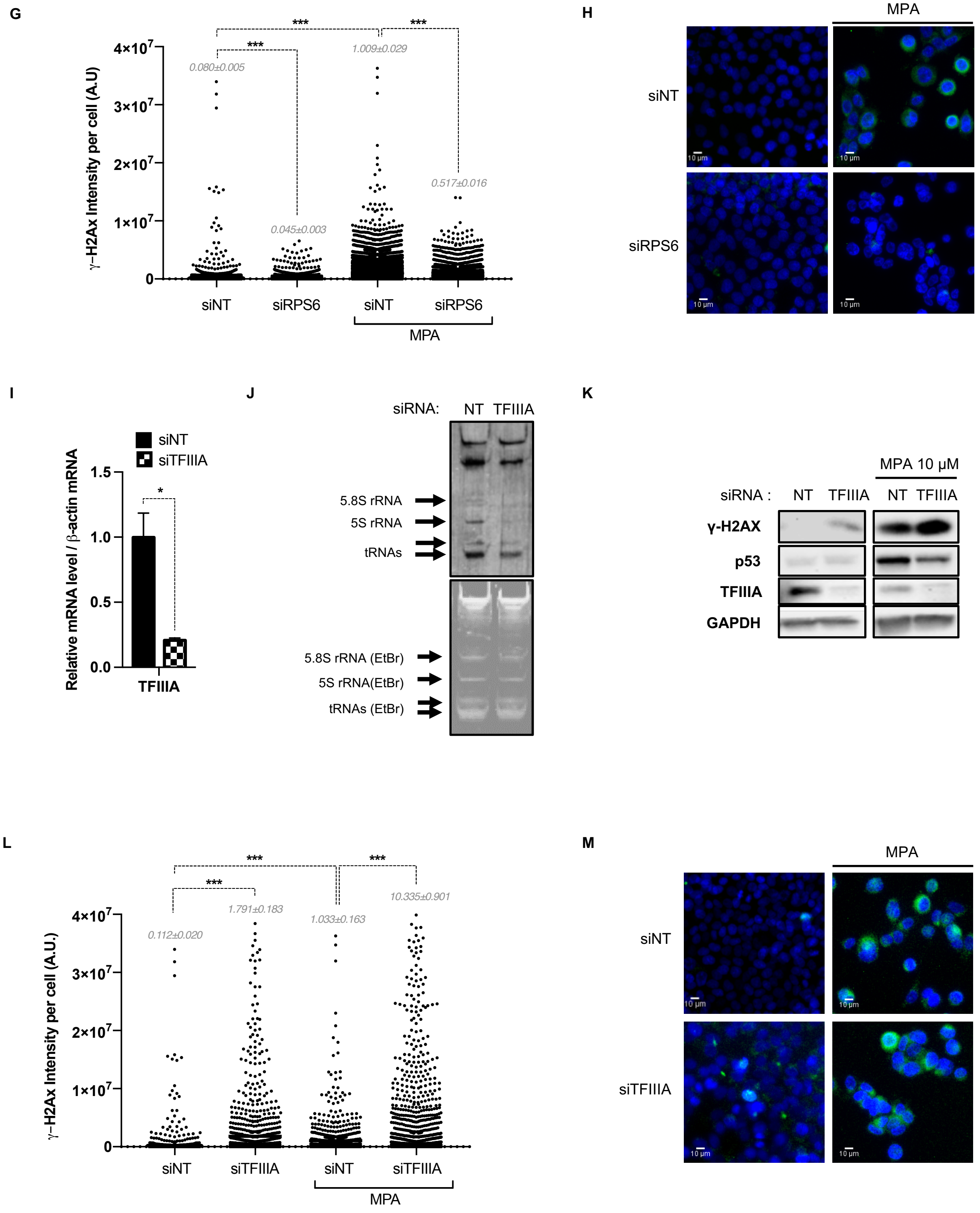

**App. Table S1. List of Antibodies**		
**Antibodies**	**Source**	**Identifier**
53BP1	Antibody Bionova	NB100-304
anti-BrDU (Flow Cytometry)	BD Biosciences	5123619
anti-α-Tubulin	Sigma	T9026
anti-γ-H2AX (Western blots)	Cell signaling	9718
anti-γ-H2AX (Immunofluorescence)	Abcam	ab22551
anti-BRCA1	Cell signaling	9010
anti-BrDU	BD Biosciences	347580
anti-Chk1	Cell signaling	2360
anti-Chk2	Cell signaling	3440
anti-Cyclin A	Santa Cruz	sc-751
anti-Cyclin D1	Cell signaling	2978
anti-Fibrillain	Santa Cruz	sc-25397
anti-GAPDH	Cell signaling	2118
anti-H2AX	Cell signaling	7631
anti-HDM2/MDM2	Abcam	ab16895
anti-IMPDH (pan antibody)	Sigma	WH0003614M1
anti-IMPDH2	Abcam	ab131158
anti-mTOR	Cell signaling	2983
Anti-p21 (Western blot)	Santa Cruz	sc-6246
Anti-p21 (Flow Cytometry)	abcam	ab109520
anti-p53	Santa Cruz	sc-6243
anti-phospho-BRCA1 (S1524)	Cell signaling	9009
anti-phospho-Chk1 (S345)	Cell signaling	2348
anti-phospho-Chk2 (T68)	Cell signaling	2197
anti-phospho-p53 (S15)	Cell signaling	9286
anti-phospho-Rb (S780)	Cell signaling	9307
anti-Rb	Cell signaling	9309
anti-RPA32/RPA2	Cell signaling	2208
anti-RPL5	Bethyl	A303-933A
anti-RPL11	Invitrogen	37-3000
anti-RPS19	abcam	ab123290
anti-TFIIIa	abcam	ab129440
anti-UBF	Santa Cruz	sc-13125

Appendix Table S2. List of siRNA and qPCR/PCR primer sequences	
**siRNA**	**Sequence**
siNT	GCATCAGTGTCACGTAATA
sip53	GCATCTTATCCGAGTGGAA
siRPL7a	CACCACCTTGGTGGAGAACAA
siRPL11	AAGGTGCGGGAGTATGAGTTA
siRPS6	TTGTAAGAAAGCCCTTAAATA
siTFIIIa	CACUAGGCAUGCUGUUGUA
**shRNA**	**Sequence**
IMPDH2	CCAAGTACATCAAAGACAAATA
IMPDH2#2	ACAGAACAGATATTTCAGTGAA
IMPDH1	GGAAGCTGCCTATCGTCAATG
**qPCR**	**Primer Sequence**
5S rRNA forward	GGCCATACCACCCTGAACGC
5S rRNA reverse	CAGCACCCGGTATTCCCAGG
28S rRNA forward	CCCAGTGCTCTGAATGTCAA
28S rRNA reverse	AGTGGGAATCTCGTTCATCC
β-actin forward	AATGTGGCCGAGGACTTTGATTGC
β-actin reverse	AGGATGGCAAGGGACTTCCTGTAA
c-MYC forward	ATTCTGCCCATTTGGGGACAC
c-MYC reverse	GTTCTCCTCCTCGTCGCAGT
IMPDH1 forward	CAGGTGTGACGTTGAAAGAGG
IMPDH1 reverse	AGCTGCTTCTGGGAATCCTTG
IMPDH2 forward	GGGAAGTGGCTCCATCTGCATT
IMPDH2 reverse	TTCCTCCATCAGCAATGACCGG
ITS1 forward	CCCGTGGTGTGAAACCTTC
ITS1 reverse	GACGAGACAGCAAACGGGAC
ITS2 forward	GTCGCTTGCCGATCAATCGC
ITS2 reverse	TCTGCGCTTAGGGGGACGGA
Firefly Luciferase forward	ACAGATGCACATATCGAGGTG
Firefly Luciferase reverse	GATTTGTATTCAGCCCATATTCG
p21 forward	TCTCAGGGTCGAAAACGGC
p21 reverse	AGAAGATCAGCCGGCGTTTG
RPL7a forward	GCTGAAAGTGCCTCCTGCGA
RPL7a reverse	CACCAAGGTGGTGACGGTGT
RPL5 forward	GGTGTGAAGGTTGGCCTGAC
RPL5 reverse	GGCACCTGGCTGACCATCAA
RPL11 forward	TCCACTGCACAGTTCGAGGG
RPL11 reverse	AAACCTGGCCTACCCAGCAC
RPS6 forward	TCTTGACCCATGGCCGTGTC
RPS6 reverse	GCGGCGAGGCACTGTAGTAT
SDH-B forward	GGATCTTGTTCCCGATTTGAGC
SDH-B reverse	TCTATGGACTGCAGATACTGCTG
TFIIIa forward	TTGTGTGTGAACATGCTGGCTGTG
TFIIIa reverse	TGAGATGAGAGGCCAAACTCCGTT

Appendix Table S3. Multiple reaction monitoring of analyzed nucleotides

Metabloite	1st transition (CE)	2nd transition (CE)	Polarity
Guanosine	282 → 150	282 → 133	Negative
IMP	347 → 78.9	347 → 96.9	Negative
dGMP	346 → 150	346 → 79	Negative
ATP	508 → 136	508 → 410	Positive
NADH	664 → 346	664 → 397	Negative
dGTP	508 → 152.2	508 → 81	Positive
dGDP	426 → 79	426 → 159	Negative
GTP	524 → 152	524 → 135	Positive
GMP	364 → 152	364 → 135	Positive
GDP	444 → 152	444 → 135	Positive
NAD	662 → 273	662 → 328	Negative
NADP	742 → 620	742 → 408	Negative
NADP	744 → 408	744 → 397	Negative
UTP	485 → 97	485 → 227	Positive
